# Lung Inflation With Hydrogen During the Cold Ischemia Phase Alleviates Lung Ischemia-Reperfusion Injury by Inhibiting Pyroptosis in Rats

**DOI:** 10.3389/fphys.2021.699344

**Published:** 2021-08-02

**Authors:** Panpan Zheng, Jiyu Kang, Entong Xing, Bin Zheng, Xueyao Wang, Huacheng Zhou

**Affiliations:** Department of Anesthesiology, The Fourth Affiliated Hospital, Harbin Medical University, Harbin, China

**Keywords:** lung transplantation, hydrogen, pyroptosis, lung inflation, cold ischemia phase

## Abstract

**Background:** Lung inflation with hydrogen is an effective method to protect donor lungs from lung ischemia-reperfusion injury (IRI). This study aimed to examine the effect of lung inflation with 3% hydrogen during the cold ischemia phase on pyroptosis in lung grafts of rats.

**Methods:** Adult male Wistar rats were randomly divided into the sham group, the control group, the oxygen (O_2_) group, and the hydrogen (H_2_) group. The sham group underwent thoracotomy but no lung transplantation. In the control group, the donor lungs were deflated for 2 h. In the O_2_ and H_2_ groups, the donor lungs were inflated with 40% O_2_ + 60% N_2_ and 3% H_2_ + 40% O_2_ + 57% N_2_, respectively, at 10 ml/kg, and the gas was replaced every 20 min during the cold ischemia phase for 2 h. Two hours after orthotopic lung transplantation, the recipients were euthanized.

**Results:** Compared with the control group, the O_2_ and H_2_ groups improved oxygenation indices, decreases the inflammatory response and oxidative stress, reduced lung injury, and improved pressure-volume (P-V) curves. H_2_ had a better protective effect than O_2_. Furthermore, the levels of the pyroptosis-related proteins selective nucleotide-binding oligomerization domain-like receptor protein 3 (NLRP3), cysteinyl aspartate specific proteinase (caspase)-1 p20, and the N-terminal of gasdermin D (GSDMD-N) were decreased in the H_2_ group.

**Conclusion:** Lung inflation with 3% hydrogen during the cold ischemia phase inhibited the inflammatory response, oxidative stress, and pyroptosis and improved the function of the graft. Inhibiting reactive oxygen species (ROS) production may be the main mechanism of the antipyroptotic effect of hydrogen.

## Introduction

The preservation of donor lungs during the cold ischemia phase (CIP) is crucial for prognosis after lung transplantation (LTx). The conventional preservation methods for donor lungs include lung preservation solution and perfusion solution, hypothermic preservation (4–8°C), and lung inflation with oxygen or air (de Perrot and Keshavjee, 2004). However, these methods are not sufficient for lung preservation. Recently, *ex vivo* lung perfusion (EVLP), as a modern technique for preserving donor lungs, was shown to be better for preserving lung function, but this method requires complicated and special equipment (Machuca et al., 2013).

The application of hydrogen for organ transplantation has been extensively studied, and hydrogen can alleviate graft injury during heart, liver, kidney, and lung transplantation (Noda et al., 2013; Kobayashi and Sano, 2019; Uto et al., 2019; Saito et al., 2020). Ventilation of donor lungs with 2% hydrogen during EVLP can improve the quality of the lung graft in rats (Noda et al., 2014). The protective effect of hydrogen on donor lungs could expand the donor pool to brain death donors and cardiac death donors and alleviate the shortage of donor lungs in rats (Zhou et al., 2013; Zhang et al., 2019). In mouse allogeneic tracheal transplantation, oral hydrogen-saturated water daily suppresses the development of mid-term obliterative airway disease (Ozeki et al., 2020). Anti-inflammatory, antioxidative, and antiapoptotic effects are mainly mechanisms that hydrogen alleviates lung injury (Kawamura et al., 2010).

Pyroptosis plays an important role in donor lung injury, and inhibiting pyroptosis could improve the quality of donor lungs (Noda et al., 2017). Lung ischemia-reperfusion injury (IRI) could be alleviated by inhibiting the selective nucleotide-binding oligomerization domain-like receptor protein 3 (NLRP3) inflammasome, which is involved in the classical pyroptosis pathway (Xu et al., 2018). Hydrogen-rich water can inhibit pyroptosis and improve acute pancreatitis by inhibiting NLRP3 inflammasome activation (Ren et al., 2014). However, the effect of hydrogen on pyroptosis in lung grafts remains unclear. In this study, we will assess the effects of lung inflation with 3% hydrogen during the CIP on pyroptosis in lung grafts.

## Materials and Methods

### Study Design

Adult male pathogen-free Wistar rats weighing 280 ± 11 g were purchased from the Second Affiliated Hospital of Harbin Medical University (Harbin, China) and were housed in individual cages with free access to food and water. The cages were placed in a temperature-controlled room with a 12 h light-dark cycle. All protocols in this study were approved by the Institutional Animal Care and Use Committee of Harbin Medical University.

**FIGURE 1:**
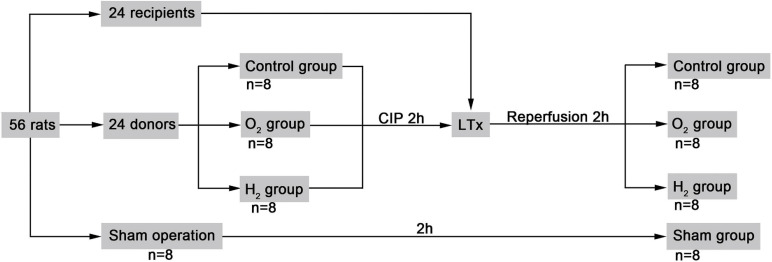
Study design. The control group, natural collapsed donor lung; The 0_2_ group, donor lung inflation with 40% oxygen; The H_2_ group, donor lung inflation with 40% oxygen and 3% hydrogen; CIP, cold ischemia phase; LTx, lung transplantation.

Fifty-six rats were randomly divided into sham group, the control group, the oxygen (O_2_) group, and the hydrogen (H_2_) group. After being harvested from donor rats, donor lungs for the control group were deflated, and those for the O_2_ group and the H_2_ group were inflated with 40% O_2_ + 60% N_2_ and 3% H_2_ + 40% O_2_ + 57% N_2_, respectively, at 10 ml/kg (LiMing Gas Corporation, Harbin, China) during the cold storage phase. The inflation gas was replaced every 20 min with an airtight injector (Agilent Corporation, California, United States) for 2 h. Then, the animals in the control group, O_2_ group, and H_2_ group underwent lung transplantation. The sham group underwent thoracotomy but no lung transplantation (Figure 1).

### Donor Harvesting

The donor rats were anesthetized by intraperitoneal injection of 60 mg/kg sodium pentobarbital, intubated by tracheotomy, and ventilated with room air (about 21% oxygen) with a tidal volume of 10 ml/kg (volume-control ventilation mode) and a rate of 40–60 breaths/min and PEEP was 0 (Model 683, Harvard Apparatus, MA, United States). Five minutes after intravenous injection of sodium heparin (1,000 U/kg) via the tail vein, the donor rats underwent thoracotomy, and the lungs were flushed from the right ventricle with 20 ml low-potassium dextran (LPD) solution prepared by Harbin Medical University according to a previous study (Pizanis et al., 2012) at a pressure of 20 cmH_2_O. Then, the donor lungs were harvested and stored at 4°C for appropriate ventilation treatment.

### Orthotopic Left LTx

The recipient rats were anesthetized and ventilated in a similar manner as the donors. A left thoracotomy was performed in the fourth intercostal space. The hilar structure of the donor lung was fully exposed under a microscope. The tidal volume was adjusted to two-thirds of that before transplantation, and then LTx was performed as described previously (Zhai et al., 2008). The tidal volume was restored, and 2 cmH_2_O positive end-expiratory pressure (PEEP) was applied after transplantation. The recipient rats were observed for 2 h. The breathing rate was adjusted to keep arterial carbon dioxide tension (PaCO_2_) within 35–45 mmHg. Anesthesia was maintained by intermittent intraperitoneal injection of pentobarbital sodium, muscle relaxation was maintained by pipecuronium bromide (0.4 mg.kg^–1^.h^–1^), and humoral balance was maintained by intravenous injection of saline (10 ml.kg^–1^.h^–1^). At the end of the experiment, the recipient rats were sacrificed by exsanguination, and the lung grafts were harvested and stored at −80°C for further analysis.

### Blood Gas Analysis

Arterial blood gas analysis was performed before transplantation (T_0_) and 3 min (T_1_), 30 min (T_2_), 60 min (T_3_), 90 min (T_4_), and 120 min (T_5_) after reperfusion (Rapid Lab 348, Bayer, Medfield, United States). At the end of the experiment, pulmonary vein blood was collected for blood gas analysis.

### Measurement of the Static Compliance of Lung Grafts

Thoracotomy was performed immediately after sacrifice, and the lungs were connected to a homemade apparatus. The static pressure-volume (P-V) curves of the lung grafts were measured to assess static compliance. The airway pressure was measured by increasing the pressure to 30 cmH_2_O and then decreasing it to 0 cmH_2_O in 5-cm stepwise intervals to evaluate lung volume, and the values were recorded after 30 s of stabilization (Meng et al., 2016).

### Histopathologic Analysis of Lung Grafts

The lung grafts were fixed in 4% paraformaldehyde, embedded in paraffin, cut into 4-μm thick sections, and stained with hematoxylin and eosin. The parameters that were evaluated included neutrophil infiltration, hemorrhage, interstitial edema, hyaline membrane formation, and airway epithelial cell damage (normal = 0, minimal change = 1, mild change = 2, moderate change = 3, and severe change = 4) (Pirat et al., 2006). A professional pathologist who was blinded to the study design evaluated all the sections and recorded the lung injury score (LIS).

### Measurement of Inflammatory Cytokines Levels in Serum and Oxidative Stress in the Graft

The upper section of the lung graft was placed in an 80°C oven for approximately 72 h until its weight stabilized, and the wet weight/dry weight (W/D) was measured. Myeloperoxidase (MPO) activity, the malondialdehyde (MDA) level, and superoxide dismutase (SOD) activity in the inferior lung graft were determined by respective kits (Jiancheng Bio-Technology, Nanjing, China). The levels of interleukin (IL)-1β and IL-18 in serum were measured with enzyme-linked immunosorbent assay (ELISA) kits (Wuhan Boster Bio-Engineering, Co., Ltd., Wuhan, China). The analysis was performed according to the manufacturer’s instructions.

### Analysis of Reactive Oxygen Species (ROS) Content in Lung Grafts

A portion of each lung graft was embedded in an optimal cutting temperature compound. Sections (8 μm thick) were obtained with a freezing microtome (Microm HR560, Thermo Fisher Scientific, United States). The sections were incubated for 30 min with 10 μM dihydroethidium (DHE) (Beyotime Biotechnology, Shanghai, China) to evaluate lung superoxide anion levels *in situ*. DHE was oxidized by superoxide anion to yield ethidium, which stains DNA with bright red fluorescence. Images were taken with a fluorescence microscope (OLYMPUS BX53M, Olympus Corporation, Japan). The fluorescence intensity was quantified by ImageJ software (v1.33, NIH, Bethesda, MD, United States).

### Detection of Pyroptosis-Related Proteins

Total proteins were extracted from lung tissue homogenates using RIPA lysis buffer (Beyotime Biotechnology, Shanghai, China). The total protein concentrations were measured using an enhanced BCA protein assay kit (Beyotime Biotechnology, Shanghai, China) according to the manufacturer’s instructions. Subsequently, the extracted protein samples were separated on 10–12.5% sodium dodecyl sulfate-polyacrylamide gel electrophoresis (SDS-PAGE). After electrophoresis, the proteins were transferred to polyvinylidene fluoride (PVDF) membranes, and the PVDF membranes were blocked with 5% fat-free milk for 2 h. Then, the membranes were incubated with primary antibodies against NLRP3 (1:1,000, Abcam, Cambridge, United Kingdom), precursors of cysteinyl aspartate specific proteinase (procaspase)-1(1:500, Wanleibio, Shenyang, China) and cysteinyl aspartate specific proteinase (caspase-1) p20 (1:500, Wanleibio, Shenyang, China), gasdermin D (GSDMD), the N-terminal of GSDMD (GSDMD-N) (1:1,000, Cell Signaling Technology, United States), and β-actin (1:1,000, ZSGB-BIO, Beijing, China) at 4°C for 24 h. After three washes, the membranes were incubated with a peroxidase-conjugated secondary antibody (1:1,000, ZSGB-BIO, Beijing, China). Finally, the immune complexes were visualized with BeyoECL Plus (Beyotime Biotechnology, Shanghai, China), and the bands were quantified with ImageJ software.

### Transmission Electron Microscopy (TEM) Analysis of Type II Alveolar Epithelial Cell Morphology

Pieces of lung grafts (1 mm^3^) were collected and fixed in 2.5% glutaraldehyde to observe the morphology of cytoplasm and nucleus. The samples were tested by a specialist at the Electron Microscope Center of Harbin Medical University who was blinded to the study design.

### Statistical Analysis

SPSS 21.0 statistical software was used for analysis, and the data are expressed as the mean values ± standard deviations or medians (interquartile ranges). Group comparisons for normal data were performed by one-way analysis of variance (ANOVA) followed by the Student-Newman-Keuls test or the Kruskal-Wallis test. Repeated measurement data were analyzed by repeated-measures ANOVA. Differences were considered statistically significant at *P* < 0.05.

## Results

### Recipient Blood Gas Analysis

**FIGURE 2:**
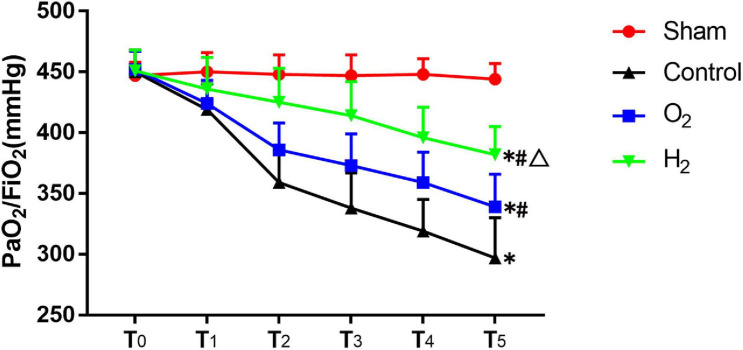
The indices of PaO_2_/FiO_2_ in each group (*n* = 8). Values are mean values ± standard deviations. T_0_–T_5_ represented the following time points: baseline before transplantation, and 3, 30, 60, 90, and 120 min after reperfusion. PaO_2_/FiO_2_, partial pressure of arterial oxygen (PaO_2_)/fraction of inspired oxygen (FiO_2_). ^∗^*P* < 0.05 vs. sham group; ^#^*P* < 0.05 vs. control group; ^△^*P* < 0.05 vs. O_2_ group.

In the sham group, partial pressure of arterial oxygen (PaO_2_)/fraction of inspired oxygen (FiO_2_) was stable. At T_5_, the PaO_2_/FiO_2_ of the control group (297 ± 33 mmHg) was lower than that of the sham group (444 ± 13 mmHg) (*P* < 0.05). The PaO_2_/FiO_2_ of the O_2_ (339 ± 27 mmHg) and H_2_ (382 ± 23 mmHg) groups was higher than that of the control group (*P* < 0.05). Furthermore, the PaO_2_/FiO_2_ of the H_2_ group was higher than that of the O_2_ group (*P* < 0.05) (Figure 2). Besides, pulmonary venous oxygen tension (PvO_2_)/FiO_2_ which is more directly reflects the pulmonary ventilation function exhibited the same trend as the PaO_2_/FiO_2_ (Table 1).

**TABLE 1:** PvO_2_/FiO_2_ and the indices of the inflammatory response and oxidative stress in each group (mean ± SD, *n* = 8).

	**PvO_2_/FiO_2_ (mmHg)**	**W/D**	**MPO (U/g)**	**IL-1β (pg/ml)**	**IL-18 (pg/ml)**	**MDA (nmol/mg prot)**	**SOD (U/mg prot)**
Sham	448 ± 15	4.5 ± 0.3	0.47 ± 0.14	47 ± 16	15 ± 5	0.79 ± 0.27	182 ± 36
Control	299 ± 41*	6.8 ± 1.1*	1.18 ± 0.28*	219 ± 51*	112 ± 29*	2.50 ± 0.52*	82 ± 26*
O_2_	348 ± 26*^#^	5.7 ± 0.8*^#^	0.94 ± 0.20*^#^	151 ± 37*^#^	74 ± 18*^#^	1.67 ± 0.47*^#^	129 ± 43*^#^
H_2_	395 ± 32*^#△^	4.9 ± 0.6^#△^	0.61 ± 0.18^#△^	90 ± 26*^#△^	44 ± 12*^#△^	0.95 ± 0.38^#△^	167 ± 34^#△^

PvO_2_/FiO_2_, pulmonary venous oxygen tension (PaO_2_)/fraction of inspired oxygen (FiO_2_);IL, Interleukin; MDA, malonaldehyde; MPO, myeloperoxidase; SOD, superoxide dismutase W/D, wet weight (W)/dry weight (D). *P < 0.05 vs. sham group; ^#^P < 0.05 vs. control group; ^△^P < 0.05 vs. O_2_ group.

### The P-V Curves of the Lung Grafts

**FIGURE 3:**
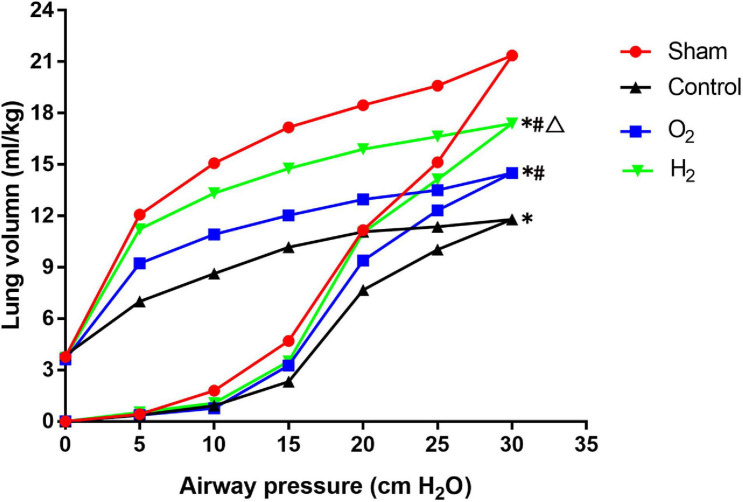
The pressure-volume (P–V) curve in the lung graft (*n* = 3). Values are means, standard deviation bars were not shown for clarity. At a pressure of 30 cmH_2_O, the lung volume in the control group was lower than the sham group (*P* < 0.05), the lung volume in O_2_ and H_2_ groups was higher than the control group (*P* < 0.05), the lung volume in the H_2_ group was higher than the O_2_ group (*P* < 0.05). ^∗^*P* < 0.05 vs. sham group; ^#^*P* < 0.05 vs. control group; ^△^*P* < 0.05 vs. O_2_ group.

At a pressure of 30 cmH_2_O, the P-V curve values of the control group (11.80 ± 0.26 ml/kg) were lower than those of the sham group (21.37 ± 1.00 ml/kg) (*P* < 0.05). The P-V curve values of the O_2_ group (14.50 ± 0.53 ml/kg) and the H_2_ group (17.40 ± 0.26 ml/kg) were higher than those of the control group (*P* < 0.05). The P-V curve values of the H_2_ group were significantly higher than those of the O_2_ group (*P* < 0.05) (Figure 3).

### The Inflammatory Response in the Recipients

The W/D ratio of the graft in the control group (6.8 ± 1.1) was higher than that of the sham group (4.5 ± 0.3) (*P* < 0.05). The W/D ratio of the graft in the O_2_ group (5.7 ± 0.8) and H_2_ group (4.9 ± 0.6) was lower than that of the control group (*P* < 0.05). The W/D ratio of the graft in the H_2_ group was lower than that of the O_2_ group (*P* < 0.05). MPO activity in the grafts and the levels of IL-1β and IL-18 in the serum exhibited a similar tendency as the W/D ratio (Table 1).

### The Oxidative Stress Response in the Recipients

SOD activity in the graft in the control group (82 ± 26 U/mg protein) was lower than that in the sham group (182 ± 36 U/mg protein) (*P* < 0.05). SOD activity in the graft in the O_2_ group (129 ± 43 U/mg protein) and the H_2_ group (167 ± 34 U/mg protein) was higher than that in the control group (*P* < 0.05). SOD activity in the H_2_ group was higher than that in the O_2_ group (*P* < 0.05). MDA levels in the graft exhibited the opposite tendency as SOD activity (Table 1).

**FIGURE 4:**
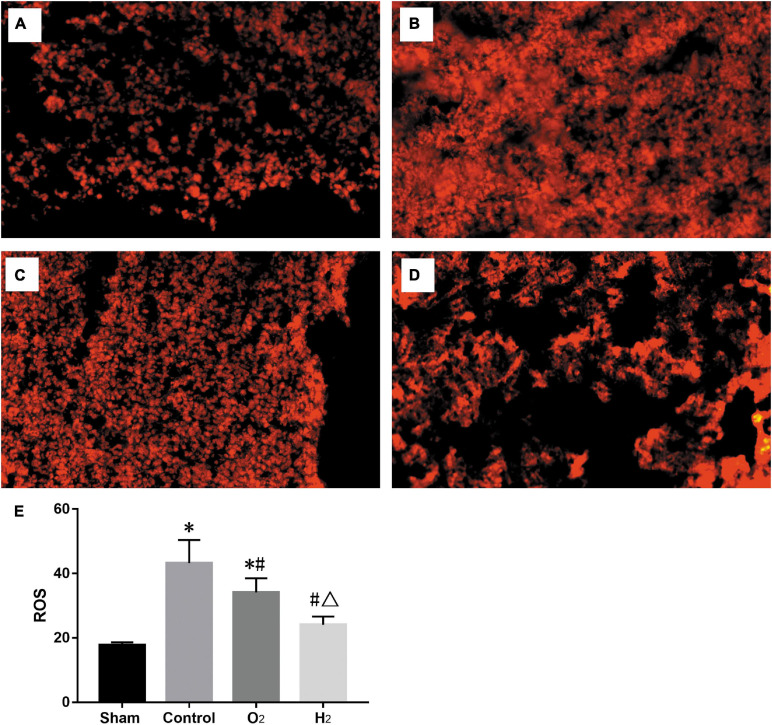
The ROS stained with dihydroethidium imaged by fluorescence microscope. (*n* = 3, original magnification, 20) **(A)** sham group; **(B)** control group; **(C)** O_2_ group; **(D)** H_2_ group; **(E)** the ROS levels in lung grafts. The ROS level in the control group was higher than the sham group (*P* < 0.05), the ROS levels in the O_2_ and H_2_ group were lower than the control group (*P* < 0.05), the H_2_ group was lower than the O_2_ group (*P* < 0.05). ^∗^*P* < 0.05 vs. sham group; ^#^*P* < 0.05 vs. control group; ^△^*P* < 0.05 vs. O_2_ group.

Similarly, the ROS level in the control group (43.2 ± 7.2) was higher than that in the sham group (17.7 ± 0.9), the ROS level in the O_2_ group (34.1 ± 4.4), and H_2_ group (24.1 ± 2.5) was lower than that in the control group, and the H_2_ group produced fewer ROS than the O_2_ group (*P* < 0.05) (Figure 4).

### Recipient Lung Injury Scores

**FIGURE 5:**
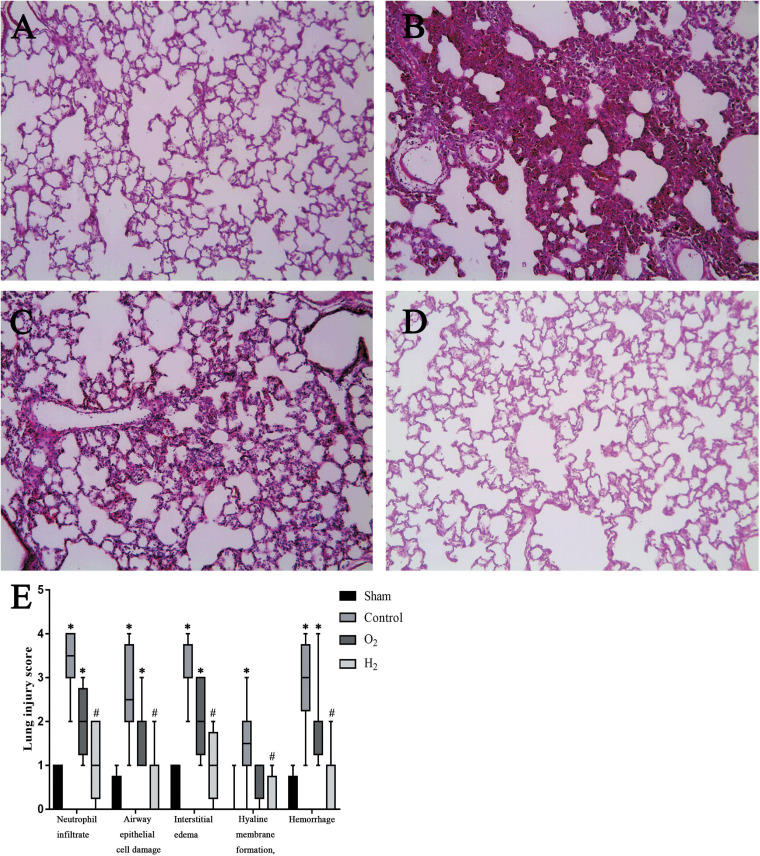
Histologic analyses of lung grafts (*n* = 8, original magnification, 40). **(A)** sham group; **(B)** control group; **(C)** O_2_ group; **(D)** H_2_ group; **(E)** Lung injury score (LIS). The LIS in the control group was higher than the sham group (*P* < 0.05), the H_2_ group was lower than the control group (*P* < 0.05). ^∗^*P* < 0.05 vs. sham group; ^#^*P* < 0.05 vs. control group; ^△^*P* < 0.05 vs. O_2_ group.

The lung grafts were almost normal in the sham group, and few pathological changes were found. However, numerous pathological changes were found in the control group, including severe interstitial edema, intra-alveolar hemorrhage, and massive neutrophil infiltration. There were fewer changes in the grafts in the O_2_ group and H_2_ group than in the control group, and hydrogen had a better therapeutic effect than O_2_. The LIS related to neutrophil infiltration in the control group [3.5(3–4)] was higher than that in the sham group [0.5(0–1)] (*P* < 0.05). The LIS related to neutrophil infiltration in the O2 group [2(1.75–2.25)] and H2 group [1(0.75–2)] was lower than that in the control group, but the difference between the control group and the O2 group was not significant. The LIS of the H2 group was lower than that of the O2 group, but the difference was not significant. Similarly, the LISs related to other criteria exhibited a similar trend as the LIS related to neutrophil infiltration (Figure 5).

### Pyroptosis-Related Data in the Recipients

**FIGURE 6:**
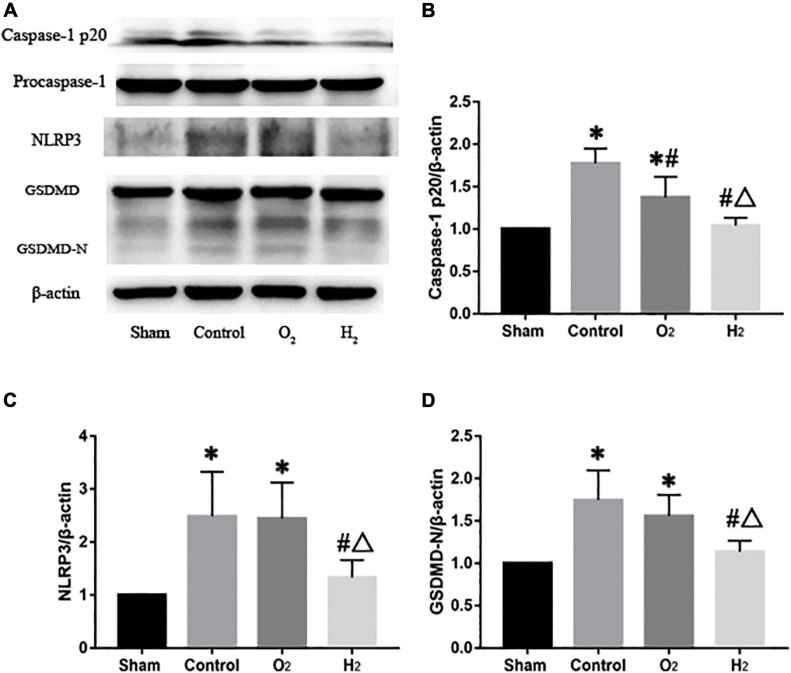
The protein expressions in the lung graft (*n* = 3). **(A)** Representative western blots and semiquantitative analysis of caspase-1 p20 **(B)**, NLRP3 **(C)**, GSDMD-N **(D)**. The expressions of caspase-1 p20, NLRP3, and GSDMD-N in the control group were higher than the sham group (*P* < 0.05), Caspase-1 p20, NLRP3, and GSDMD-N in the O_2_ group were lower than the control group, but the difference of NLRP3 and GSDMD-N had no statistically significant, Caspase-1 p20, NLRP3, and GSDMD-N in the H_2_ group were lower than the control group and the O_2_ group (*P* < 0.05). ^∗^*P* < 0.05 vs. sham group; ^#^*P* < 0.05 vs. control group; ^△^*P* < 0.05 vs. O_2_ group.

The levels of NLRP3 in the lung grafts were higher in the control group (2.5 ± 0.8) than that in the sham group (*P* < 0.05). The levels of NLRP3 in the lung grafts in the O_2_ group (2.4 ± 0.7) and H_2_ group (1.3 ± 0.3) were lower than those in the lung grafts in the control group, but the difference between the control group and the O_2_ group was not significant. In the H_2_ group, the level of NLRP3 was lower than that in the O_2_ group (*P* < 0.05). The levels of caspase-1 p20 and GSDMD-N exhibited a similar tendency as the level of NLRP3. The levels of procaspase-1 and GSDMD were not different between the groups (Figure 6).

### Electron Microscopy Analysis of Morphological Changes in Type II Alveolar Epithelial Cells

**FIGURE 7:**
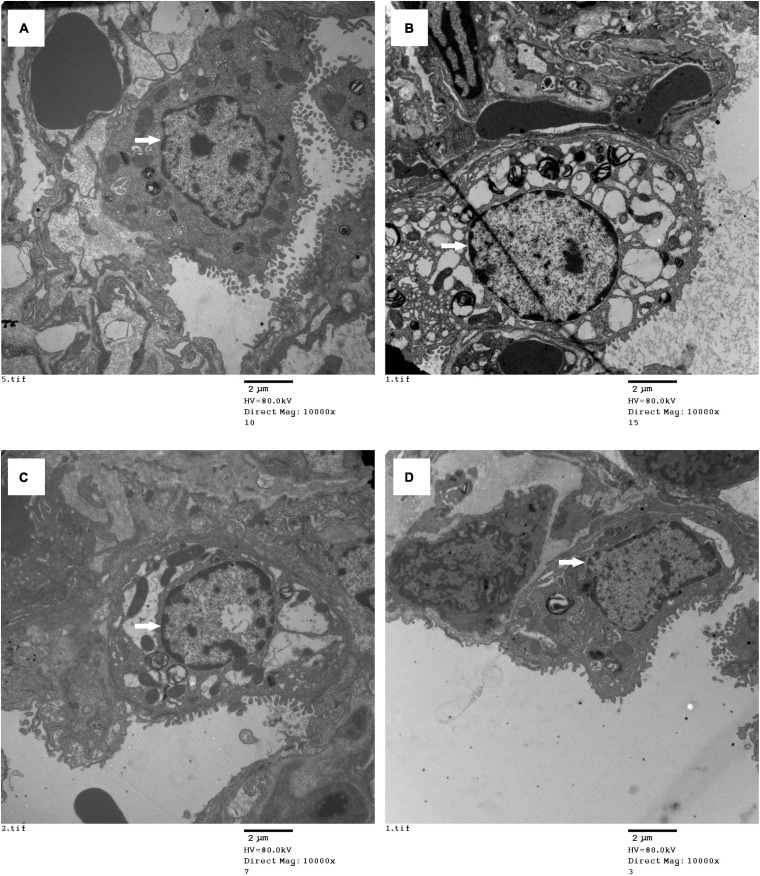
Type II alveolar epithelial cell morphology was imaged with electron microscopy (original magnification, 10,000). **(A)** sham group; **(B)** control group; **(C)** O_2_ group; **(D)** H_2_ group. The type II alveolar epithelial cell morphology was normal in the sham group, the swollen cell and nuclear pyknosis were observed in the control group, the changes were improved in the O_2_ group, the cell had fewer changes in the H_2_ group. ^∗^*P* < 0.05 vs. sham group; ^#^*P* < 0.05 vs. control group; ^△^*P* < 0.05 vs. O_2_ group.

The ultrastructural changes in the lung grafts were observed by TEM. In the sham group, the type II alveolar epithelial cells were almost normal. However, severely swollen cytoplasmic and nuclear pyknosis was found in the control group, there were fewer changes in the O_2_ group than in the control group, and few changes were found in the H_2_ group (Figure 7).

## Discussion

In this study, the expression of pyroptosis-related proteins, including NLRP3, caspase-1 p20, and GSDMD-N, was increased, and pyroptotic type II alveolar epithelial cells were found in lung grafts by electron microscopy. These data indicate that pyroptosis occurred in lung grafts. When the donor lung was inflated with 40% oxygen during the CIP, oxygenation and static compliance were improved, and oxidative stress indices, including MDA levels and SOD activity, were improved. Inflammatory response indices, including the W/D ratio, MPO activity, and the levels of IL-1β and IL-18, were decreased. The morphology of type II alveolar epithelial cells was improved, and the expression of caspase-1 p20 was decreased, which is the result of the combination of oxygen and lung inflation. When oxygen was combined with 3% hydrogen, the above indices related to the inflammatory response and oxidative stress were further improved, the expression of caspase-1 p20, NLRP3, and GSDMD-N was decreased.

In donor lungs, the application of hydrogen in the cold storage phase includes lung inflation and the use of hydrogen-rich preservation solution (Pizanis et al., 2012; Xu et al., 2018). Liu et al. (2015) found that lung inflation with 3% hydrogen during the cold ischemia phase alleviated lung graft injury. Takahashi et al. (2017) found that immersing lungs of rats in hydrogen-rich saline attenuated lung ischemia-reperfusion injury (IRI) by decreasing inflammatory cytokine levels and improving pulmonary function. Compared with the use of hydrogen-rich preservation solution, lung inflation with hydrogen is simpler and easier and does not require additional equipment. The optimal concentration of hydrogen has not been determined. Liu et al. (2017) found that inhalation of 22 and 41.6% hydrogen had a better therapeutic effect on chronic obstructive pulmonary disease. Chen X found that the protective effects of 55–75% hydrogen on *in vitro* neuronal mechanical injury were dose-dependent (Chen et al., 2018). In this study, 3% hydrogen was chosen because of its safety, and because it could alleviate lung IRI in our previous experiments (Liu et al., 2015).

Lung inflation is an effective method for preserving the donor lung. Kao et al. (2004) found that static lung inflation with a mixture of room air and 5% CO_2_ attenuated IRI by decreasing the capillary filtration coefficient and protein concentration in bronchoalveolar lavage fluid. Fukuse et al. (1999) found that lung inflation with room air exerted protective effects through both lung inflation and oxygen. Inflation of the alveoli can prevent mechanical damage during reexpansion, and oxygen can attenuate lung injury through maintaining aerobic metabolism (Fukuse et al., 1999). Weder et al. (1991) thought that lung preservation with 100% oxygen inflation appears superior to inflation with room air. However, Fukuse et al. (2001) thought hyperoxygenation (50 or 100% oxygen) induced mitochondrial dysfunction and increased lipid peroxidation. Therefore, 40% oxygen was chosen for lung inflation in the oxygen group. To further improve the treatment of lung inflation with 40% oxygen, 3% hydrogen was added on 40% oxygen (Fukuse et al., 2001). Compared with lung inflation with toxic hydrogen sulfide and carbon monoxide, lung inflation with 3% hydrogen is safer and less harmful (Fukuse et al., 2001). In this study, compared with natural collapse of the lung, lung inflation with 40% O_2_ improved lung graft function, decreased the inflammatory response and oxidative stress, and attenuated lung IRI. Lung IRI was further ameliorated in the hydrogen group, indicating that hydrogen strengthened the protective effect of lung inflation with 40% O_2_, which is consistent with our previous studies (Liu et al., 2015).

Severe IRI can lead to primary graft dysfunction. Oxidative stress and the inflammatory response play an important role in lung IRI (de Perrot et al., 2003; Ng et al., 2006). Hydrogen has anti-inflammatory and selective antioxidant effects. Kawamura et al. (2010) found that inhaled hydrogen inhibited the production of proinflammatory mediators and reduced graft lipid peroxidation. In this experiment, lung inflation with hydrogen during the CIP decreased inflammatory indices, including the levels of IL-1β and IL-18 in the serum, the W/D ratio, and MPO activity in grafts, and inhibited oxidative stress, including by increasing SOD activity and reducing MDA and ROS levels. Our previous experiment also revealed that lung inflation with hydrogen could inhibit the inflammatory factors TNF-α and IL-8 (Liu et al., 2015). These protective effects of hydrogen might be achieved through inhibition of the p38 mitogen-activated protein kinase (MAPK) and nuclear factor-kappa B (NF-κB) signaling pathways (Zhang et al., 2018).

Pyroptosis is a form of inflammatory programmed cell death mediated by GSDMD. Moderate pyroptosis can act as a self-protective mechanism against infection, and severe pyroptosis can aggravate lung injury (Man et al., 2017). Fei et al. (2020) found that severe pyroptosis was present in a mouse model of lung IRI and inhibited pyroptosis via the alveolar macrophage pathway to ameliorate lung IRI with recombinant high-mobility group box 1. Xu et al. (2018) found that in a mouse model of lung IRI, pyroptosis in the lung was more severe 2 h after reperfusion than 1 or 6 h after reperfusion. Therefore, 2 h of reperfusion was used in this experiment. Electron microscopy and analysis of pyroptosis-related protein expression revealed that NLRP3 inflammasome-mediated pyroptosis was involved in lung IRI.

Ion flux, ROS, and lysosome rupture are the main pathways to activate the NLRP3 inflammasome (de Zoete et al., 2014). ROS, as second messengers, are considered important for the activation of the NLRP3 inflammasome. Qiu et al. (2017) found that ROS activated the NLRP3 inflammasome, which triggered caspase-1-dependent pyroptosis in myocardial IRI in diabetic rats. Some studies have confirmed that hydrogen inhibits NLRP3 inflammasome activation and reduces pyroptosis in the brain and lung injury (Shao et al., 2016; Zou et al., 2019). In this experiment, lung inflation with 3% hydrogen during the CIP decreased ROS levels and inhibited pyroptosis-related indices, including the expression of NLRP3, caspase-1 p20, and GSDMD-N. Inhibiting ROS production might be the main mechanism of the antipyroptotic effect of hydrogen. Coincidentally, Ren JD found that molecular hydrogen inhibited lipopolysaccharide-triggered NLRP3 inflammasome activation in macrophages by targeting mitochondrial ROS (Ren et al., 2016).

This experiment had the following limitations. First, lung inflation for 2 h and reperfusion for 2 h after transplantation might be too short, the long-term effects of hydrogen on lung IRI need to be further studied. Second, we found that pyroptosis occurred in lung grafts 2 h after transplantation. However, the regulatory mechanism of pyroptosis was not elucidated. Third, only the classical NLRP3 inflammasome-mediated pyroptosis pathway was assessed, and other classical and non-classical pyroptosis pathways need to be further studied. Fourth, this study only examined the therapeutic effect of 3% hydrogen, and the effects of high-concentration hydrogen need to be further studied. Fifth, the donor lungs of the control group weren’t inflated. Sixth, reactive oxygen species, the lung compliance, and the expressions of pyroptosis-related proteins were only measured in 3 animals.

In conclusion, pyroptosis occurred in lung grafts, and lung inflation with 3% hydrogen during the cold ischemia phase had anti-inflammatory, antioxidant, and antipyroptotic effects during lung transplantation. Preventing pyroptosis might be one of the mechanisms by which hydrogen alleviates lung IRI. Inhibiting ROS production might be the main mechanism of the antipyroptotic effect of hydrogen.

## Data Availability Statement

The original contributions presented in the study are included in the article/Supplementary Material, further inquiries can be directed to the corresponding author/s.

## Ethics Statement

The animal study was reviewed and approved by The Fourth Affiliated Hospital of Harbin Medical University.

## Author Contributions

PZ, JK, and HZ designed the study. PZ, JK, and EX performed the study. XW, BZ, and HZ analyzed the data. PZ drafted the manuscript. JK and HZ revised the manuscript. All authors contributed to the manuscript and approved the submitted version.

## Conflict of Interest

The authors declare that the research was conducted in the absence of any commercial or financial relationships that could be construed as a potential conflict of interest.

## Publisher’s Note

All claims expressed in this article are solely those of the authors and do not necessarily represent those of their affiliated organizations, or those of the publisher, the editors and the reviewers. Any product that may be evaluated in this article, or claim that may be made by its manufacturer, is not guaranteed or endorsed by the publisher.
