# Supplementary material for: Lung Inflation With Hydrogen During the Cold Ischemia Phase Alleviates Lung Ischemia-Reperfusion Injury by Inhibiting Pyroptosis in Rats
Source: Front Physiol. 2021 Aug 2;12:699344. doi: 10.3389/fphys.2021.699344 (PMC8365359; doi:10.3389/fphys.2021.699344)
Supplement: Supplementary file 1 [file Table_1.DOC]

Table 1. The indices of blood gas analysis in each group (mean ± SD, n = 8)

|  | Group | T_0_ | T_1_ | T_2_ | T_3_ | T_4_ | T_5_ |
| --- | --- | --- | --- | --- | --- | --- | --- |
| PaO_2_/FiO_2_  (mmHg) | Sham | 447 ± 11 | 450 ± 16 | 448 ± 16 | 447 ± 17 | 448 ± 13 | 444 ± 13 |
|  | Control | 450 ± 18 | 419 ± 21^*^ | 359 ± 27^*^ | 338 ± 29^*^ | 319 ± 26^*^ | 297 ± 33^*^ |
|  | O_2_ | 452 ± 15 | 424 ± 19 | 386 ± 22^*^ | 373 ± 26^*^ | 359 ± 25^*#^ | 339 ± 27^*#^ |
|  | H_2_ | 451 ± 17 | 436 ± 26 | 425 ± 28^#△^ | 414 ± 28^#△^ | 396 ± 25^*#△^ | 382 ± 23^*#△^ |
| pH value | Sham | 7.40 ± 0.01 | 7.38 ± 0.02 | 7.41 ± 0.04 | 7.40 ± 0.03 | 7.41 ± 0.03 | 7.40 ± 0.02 |
|  | Control | 7.39 ± 0.03 | 7.40 ± 0.04 | 7.32 ± 0.06^*^ | 7.29 ± 0.06^*^ | 7.24 ± 0.08^*^ | 7.19 ± 0.09^*^ |
|  | O_2_ | 7.38 ± 0.02 | 7.39 ± 0.03 | 7.35 ± 0.06 | 7.33 ± 0.05 | 7.30 ± 0.07^*^ | 7.25 ± 0.05^*^ |
|  | H_2_ | 7.40 ± 0.03 | 7.39 ± 0.03 | 7.37 ± 0.04 | 7.36 ± 0.05^#^ | 7.36 ± 0.05^#^ | 7.35 ± 0.07^#△^ |
| BE value | Sham | 0.07 ± 0.02 | 0.09 ± 0.04 | 0.08 ± 0.03 | 0.09 ± 0.04 | 0.09 ± 0.05 | 0.08 ± 0.04 |
|  | Control | 0.08 ± 0.03 | 0.08 ± 0.04 | 1.85 ± 0.43^*^ | 2.93 ± 0.74^*^ | 3.59 ± 0.71^*^ | 4.26 ± 0.81^*^ |
|  | O_2_ | 0.09 ± 0.04 | 0.09 ± 0.06 | 1.34 ± 0.60^*^ | 2.26 ± 0.68^*^ | 2.70 ± 0.53^*#^ | 3.35 ± 0.55^*#^ |
|  | H_2_ | 0.08 ± 0.03 | 0.07 ± 0.03 | 0.78 ± 0.52^*#^ | 1.26 ± 0.51^*#△^ | 1.79 ± 0.64^*#△^ | 2.29 ± 0.68^*#△^ |

T_0_-T_5_ represented the following time points: baseline before transplantation, and 3 min, 30 min, 60 min, 90 min, and120 min after reperfusion. BE, base excess; PaCO_2_, arterial carbon dioxide tension; PaO_2_/FiO_2_, partial pressure of arterial oxygen (PaO_2_)/fraction of inspired oxygen (FiO_2_). ^*^P < 0.05 vs sham group; ^#^P < 0.05 vs control group; ^△^P < 0.05 vs O_2_ group.
