# Supplementary material for: Lung Inflation With Hydrogen During the Cold Ischemia Phase Alleviates Lung Ischemia-Reperfusion Injury by Inhibiting Pyroptosis in Rats
Source: Front Physiol. 2021 Aug 2;12:699344. doi: 10.3389/fphys.2021.699344 (PMC8365359; doi:10.3389/fphys.2021.699344)
Supplement: Supplementary file 2 [file Table_2.DOC]

Table 2. The indices of pulmonary vein blood gas analysis in each group (mean ± SD, n = 8)

|  | PvO_2_/FiO_2_  (mmHg) | pH value | BE value |
| --- | --- | --- | --- |
| Sham | 448 ± 15 | 7.40 ± 0.03 | 0.08 ± 0.03 |
| Control | 299 ± 41^*^ | 7.20 ± 0.10^*^ | -4.24 ± 0.69^*^ |
| O_2_ | 348 ± 26^*#^ | 7.26 ± 0.06^*#^ | -3.20 ± 0.65^*#^ |
| H_2_ | 395 ± 32^*#△^ | 7.38 ± 0.09^#△^ | -2.17 ± 0.72^*#△^ |

BE, base excess; PaCO_2_, arterial carbon dioxide tension; PvO_2_/FiO_2_, pulmonary venous oxygen tension (PaO_2_)/fraction of inspired oxygen (FiO_2_). ^*^P < 0.05 vs sham group; ^#^P < 0.05 vs control group; ^△^P < 0.05 vs O_2_ group.
